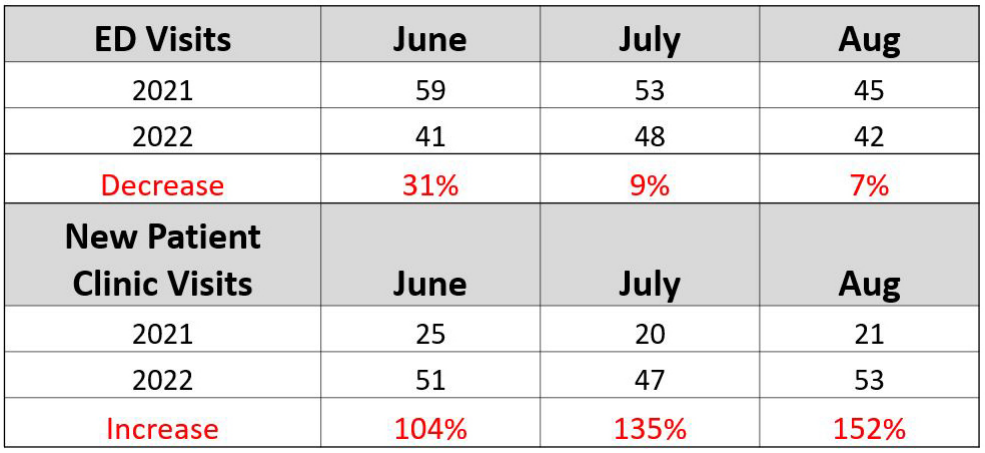# 733 Development of TeleBurn Utilizing Hospital Based EMR and Stroke Robot Photography

**DOI:** 10.1093/jbcr/irad045.208

**Published:** 2023-05-15

**Authors:** Nicole Bernal, Beth McGuire, Laura Pezzopane, Susanna Skornicka

**Affiliations:** The Ohio State University Wexner Medical Center, Columbus, Ohio; The Ohio State University Wexner Medical Center, Columbus, Ohio; The Ohio State University Wexner Medical Center, Columbus, Ohio; The Ohio State Wexner Medical Center, Columbus, Ohio; The Ohio State University Wexner Medical Center, Columbus, Ohio; The Ohio State University Wexner Medical Center, Columbus, Ohio; The Ohio State University Wexner Medical Center, Columbus, Ohio; The Ohio State Wexner Medical Center, Columbus, Ohio; The Ohio State University Wexner Medical Center, Columbus, Ohio; The Ohio State University Wexner Medical Center, Columbus, Ohio; The Ohio State University Wexner Medical Center, Columbus, Ohio; The Ohio State Wexner Medical Center, Columbus, Ohio; The Ohio State University Wexner Medical Center, Columbus, Ohio; The Ohio State University Wexner Medical Center, Columbus, Ohio; The Ohio State University Wexner Medical Center, Columbus, Ohio; The Ohio State Wexner Medical Center, Columbus, Ohio

## Abstract

**Introduction:**

Our burn center is located in a state that takes referrals from rural areas up 160 miles away. Transfer to our often at capacity emergency department for burn wound care and to be discharged is an expense to the patient and the medical system.

Using existing transfer center nurses, the EMR system, and hospitals connected in the stroke network hypothesized we could create a TeleBurn program without additional equipment or resources. This was developed to expedite patient care and move the burn evaluation for smaller burns to the outpatient clinic from the emergency department. Calls were covered by the on-call trauma attending.

**Methods:**

The TeleBurn program used 27 care connect hospitals in our stroke network. Calls were received through transfer center Figure 1. If the call was one where the physician was asking for opinion or possible follow up, the stroke robot was brought into the room, photographs taken and uploaded to EMR. A page/EMR message alerted call trauma attending physician. If the patient’s burn were small, they were set up for follow-up in clinic the next day in designate protected slots, if they needed surgery, admission for pain or wound care transfer, was arranged. The transfer center sent clinic staff message through EMR.

**Results:**

Compared to previous year there was reduction of 3-10 ED visits per month and doubled new clinic visits in those same months. During the three-month pilot period, only one patient required admission when seen in clinic. Table 1. Our no show rate for protected slots was higher at 33% compared to no show rate of 12% for scheduled burn patients.

**Conclusions:**

We demonstrated that TeleBurn triage can be done in a HIPPA compliant manor without having to purchase a new technology or platform. The development of a process with pictures and guaranteed followed up gave both the on call physician and transferring physician the confidence not to transfer a patent in middle of the night. More work is needed to determine why the no-show rate is high and how to adjust appointments to correct.

**Applicability of Research to Practice:**

It is feasible to create a successful system that uses your EMR and limits additional steps for transferring physicians and on-call surgeon. Real time communication was utilized with the team to help vet issues quickly and allow for rapid feedback.